# The Therapeuatic Effect of Endostar on Soft Carotid Plaque Neovascularization in Patients with Non-small Cell Lung Cancer

**DOI:** 10.1038/srep08956

**Published:** 2015-03-10

**Authors:** Zhaoxia Pu, Yao Wang, Ying Zhang, Jing Huang, Yurong Hong, Huiliao He, Chunmei Liu, Shuyuan Chen, Paul A. Grayburn, Pintong Huang

**Affiliations:** 1 Department of Ultrasound, the Second Affiliated Hospital of Zhejiang University School of Medicine, Zhejiang 310009, China; 2 Department of Ultrasound, the Second Affiliated Hospital of Wenzhou Medical University, Zhejiang 325027, China; 3 Baylor Heart & Vascular Institute, Baylor University Medical Center, 621 N. Hall St., Suite H030 Dallas, Texas 75226, USA

## Abstract

The purpose of this study was to investigate the effect of the angiogenesis inhibitor Endostar on carotid plaque neovascularization in patients with non-small cell lung cancer (NSCLC) using contrast-enhanced ultrasound (CEUS). Ninety-one patients who had NSCLC with soft carotid plaques were selected for treatment either with the NP regimen (vinorelbine + cisplatin) (43 patients) or with the ENP regimen (Endostar + NP) (48 patients). Plaque thickness and neovascularization of the plaque were assessed before and at 1 month after treatment using CEUS. Enhanced intensity (EI) of CEUS was used for quantification of plaque neovascularization. There was no significant changes in any group in thickness of plaque between recruitment and 1 month after treatment (*P* > 0.05 for all). There was no significant change in the EI of plaque in the controls or NP groups at 1 month after treatment (*P* > 0.05), while EI in the ENP group was significantly reduced at 1 month after treatment (*P* < 0.01) and significantly lower than that in the controls or NP group at 1 month after treatment (*P* < 0.001 both). This study indicates that carotid soft plaque neovascularization in patients with NSCLC can be reduced by anti-angiogenesis treatment.

Plaque neovascularization is associated with plaque formation, development, instability and clinical symptoms^1^,^2^,^3^,^4^,^5^. These newly formed microvessels are immature and fragile and thus may induce plaque hemorrhage and rupture, which is an important mechanism of plaque instability^6^,^7^. These findings have stimulated a search for potential treatments with potent angiogenesis inhibitors to reduce plaque neovascularization and progression. Some studies have shown that the lipid-lowering drug atorvastatin inhibits the formation and development of neovascularization in human carotid plaques^8^,^9^. Moreover, some studies have demonstrated that the angiogenesis inhibitor Endostatin can inhibit atherosclerotic plaques and neovascularization in animal models^10^,^11^. Although the concept of an “antiangiogenic” strategy in the treatment of patients with vascular disease, and a framework for further preclinical evaluation of such therapy was raised by Brendan Doyle in 2007^12^. To our knowledge, so far there are no related papers published.

The angiogenesis inhibitor Endostar, a modified recombinant human endostatin, can inhibit tumor endothelial cell proliferation, angiogenesis, and tumor growth. It is reported that the addition of rh-endostatin to gemcitabine plus cisplatin chemotherapy for first-line treatment of NSCLC can improve objective response and survival^13^. The addition of an anti-neovascular drug to a standard chemotherapy regimen for patients with non-small cell lung cancer and carotid atheroma provided an opportunity to study its effect on plaque neovascularization. In this study we investigate the effect of the angiogenesis inhibitor Endostar, on carotid plaque neovascularization in patients with non-small cell lung cancer (NSCLC) treated with Endostar combined with vinorelbine and cisplatin (NP) regimen. The effectiveness was evaluated by contrast-enhanced ultrasound (CEUS), which allows real-time assessment of the response to anti-atherosclerotic therapies. Our previous study had indicated that the enhancement index (EI), a quantitative measurement of CEUS intensity, had a good correlation with microvessel density, which reflects neovascularization within the plaque^14^.

## Results

### Patient characteristics

**Figure 1:**
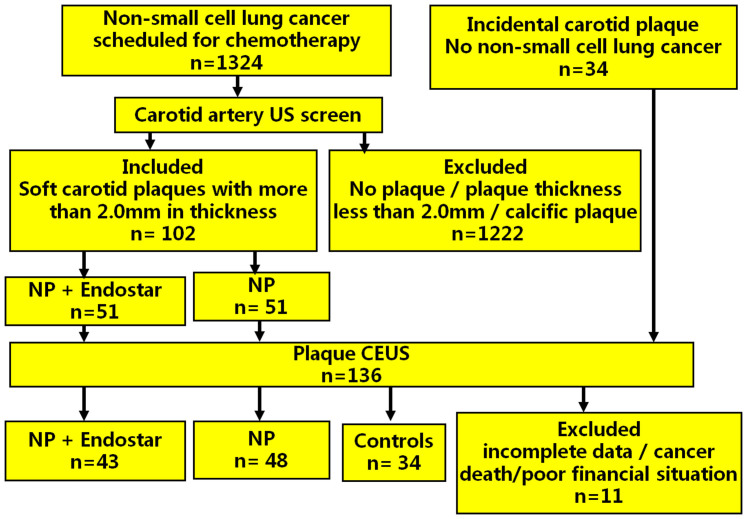
Flowchart of patient selection.

Four cases in the ENP group and 3 cases in the NP group were excluded because of missing data. One case in the ENP group was excluded because of cancer death during treatment. Three cases in the ENP group were excluded because their poor financial situation precluded them from receiving Endostar treatment. The remaining 43 cases in the ENP group and 48 cases in the NP group were enrolled in this study (Figure 1). There were no significant differences among the three groups for mean age, gender, EF, SBP, DBP and blood lipid levels [total cholesterol (TC), total triglyceride (TG), low-density lipoprotein (LDL), and high-density lipoprotein (HDL))] (*P* > 0.05 for all) (Table 1). The ratio of stage II, stage III, stage IV (stage II: 6.25% vs 6.98%; stage III: 62.5% vs 62.79%; stage IV: 31.25% vs 30.23%) were not significant difference between NP and ENP group (*P* > 0.05 for all).

### Effect of Endostar on blood lipid levels, plaque size and neovascularization

**Figure 2:**
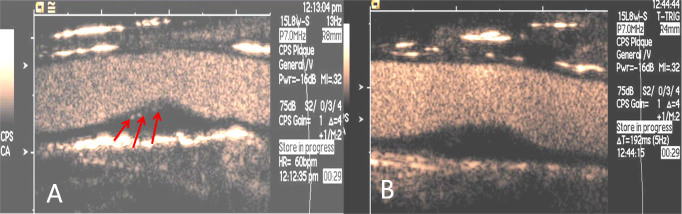
CEUS demonstrated that the plaque enhanced with peripheral punctuate enhancement before treatment (red arrows) (A) and no enhancement at one month after anti-angiogenic treatment (B).

**Figure 3:**
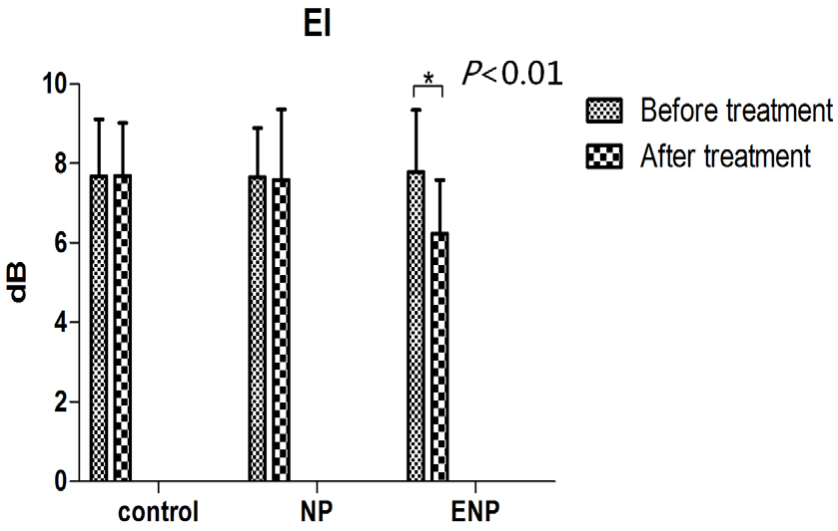
In the ENP group, the EI of the plaque at one month after treatment was significantly lower than at baseline (*P* < 0.01).

There were no significant difference in EI and thickness of plaque among the three groups at baseline (*P* > 0.05). No significant changes were found in any group for ejection fraction (EF), systolic blood pressure (SBP), diastolic blood pressure (DBP), TC, TG, LDL, HDL and the plaque thickness at 1 month after treatment compared to baseline (P > 0.05 for all) (Table 1). In the ENP group, CEUS demonstrated that some plaques enhanced with peripheral punctuate enhancement at baseline (C) and no enhancement at one month after anti-angiogenic treatment (Figure 2), the EI of the plaque at one month after treatment was significantly lower than at baseline (*P* < 0.01) (Figure 3). No significant change in EI in the plaque was found in the controls or the patients not receiving the anti-neovascular drug (*P* > 0.05 for both). The value of the EI of plaque in the ENP group was significantly lower than that in the NP group and the controls at 1 month after treatment (*P* < 0.001 for both) (Table 2).

## Discussion

The addition of an anti-neovascular drug to a standard chemotherapy regimen for some patients with lung cancer and carotid atheroma provided an opportunity to study the effect of the anti-neovascular drug on plaque neovascularization. Endostar, a novel recombinant human endostatin approved in China, inhibits tumor proliferation and metastases as a strong inhibitor of angiogenesis^15^,^16^,^17^. Endostar has been demonstrated to improve overall and progression-free survival when combined with first-line chemotherapy in patients with advanced NSCLC^18^,^19^,^20^,^21^. Endostar has recently been studied for inhibition and reduction of plaque neovascularization in animal models^11^,^22^, but no study in human patients has been published.

Contrast-agent microbubbles are purely intravascular tracers and are used for vascular ultrasound imaging with high spatial and temporal resolution^23^. More recently, contrast-enhanced ultrasound imaging has been proposed for imaging carotid plaque^24^,^25^,^26^. Feinstein et al.^27^ first described contrast-agent enhancement within carotid plaques and attributed this to plaque neovascularization. Our previous animal study indicated that the quantitative index of EI of CEUS had a good correlation with microvessel density, with good intraclass correlations for inter- and intra-observer agreement for EI in animal^14^ and human studies^26^, which reflects neovascularization within the plaque. In the present study, we performed direct visualization of neovascularization of carotid plaques by CEUS imaging to evaluate the inhibitory effect of Endostar on neovascularization. We selected patients with NSCLC because Endostar is commonly used in this type of cancer in combination with the standard NP regimen. Patients on the ENP regimen (with additional Endostar) showed a decrease in plaque enhancement at one month after treatment as compared with the NP regimen and the control groups, although plaque thickness did not change significantly, This result indicates that Endostar could inhibit neovascularization within carotid plaque, although it does not reduce the thickness of the plaque in the short-term; long-term observation will be needed to confirm duration of effect. In this study, blood lipid levels (total cholesterol (TC), total triglyceride (TG), low-density lipoprotein (LDL), and high-density lipoprotein (HDL)) in the controls were not significantly different among the three groups at baseline and no significant changes were found at 1 month after treatment). Though it has been reported that therapies targeting vascular endothelial growth factor (VEGF) were associated with hypertension, cardiotoxicity, and thromboembolic events^28^,^29^, no side effects occurred in the NP group in our observation period. The blood pressure and ejection fraction of each patient did not change significantly in the ENP group at 1 month after treatment compared to baseline. One patient in the ENP group died during treatment because of cancer related problems.

To our knowledge, this is the first clinical report investigating the inhibitory potential of anti-angiogenesis treatment on plaque neovascularization. We initially intended to allocate patients randomly to treatment with and without additional Endostar but the way this drug is funded in our country (the patients have to pay for the additional treatment themselves, though the routine regimen is government-funded) meant that the patient's financial situation determined whether or not they could receive Endostar. Thus, three patients randomized to the ENP group had to be excluded due to their poor financial situation. This is one reason for the asymmetry in the patient numbers between the two treatment groups. The control group was selected from patients attending for routine health screening, which is popular in our country.

Plaque neovascularization is emerging as a key component of unstable (vulnerable) plaques. Thus, detection of plaque neovascularization is an important prognostic factor that has been shown to correlate with the risk of cerebrovascular events. The potential to control plaque neovascularization, either directly using anti-neovascular dugs such as VEGF inhibitors, or indirectly using drugs that reduce the hypoxic components of plaque, such as the statin drugs, is intriguing and should be the basis for future studies.

Our study has several limitations. We did not assess the effect of Endostar treatment on intraplaque neovascularization on histology because our patients suffered from lung cancer and therefore carotid endarterectomy was not performed; however, Shah et al. have reported a good correlation between carotid CEUS of intraplaque neovascularization and a semiquantitative histological score on surgical specimens^30^. This study lacks a patient group with Endostar treatment only. We could not evaluate the long-term efficacy of Endostar on carotid plaques because many of our patients had limited survival time.

This study has shown that plaque neovascularization can be reduced by anti-angiogenesis treatment; this may provide a new approach for future treatment of atheroma. Plaque neovascularization may also become an important marker for assessing the results of anti-atherosclerotic therapies. CEUS imaging could be used to monitor this phenomenon inexpensively in real time in the clinical setting.

## Methods

### Patients

A total of 1324 patients with NSCLC confirmed on pathological analysis of image-guided biopsies were recruited to this study from those admitted to the Second Affiliated Hospital of Zhejiang University School of Medicine and the Second Affiliated Hospital of Wenzhou Medical University between April 2007 and March 2014. Informed consent was obtained from all patients before their examination, and the local ethics committee and institutional review board of the Second Affiliated Hospital of Zhejiang University School of Medicine and the Second Affiliated Hospital of Wenzhou Medical University approved this prospective study. The methods in this study were performed in accordance with approved guidelines. No incentives, financial or other, were offered to them. All patients were screened with B-mode carotid artery ultrasound examination. Inclusion criteria were patients with NSCLC scheduled for chemotherapy; at least one soft plaque (less echogenic than the surrounding adventitia, in the absence of any calcification) with a thickness greater than 2.0 mm; no prior treatment with lipid-lowering or hypoglycemic drugs (to avoid inhibition of formation and development of neovascularization in human carotid plaques)^8^,^9^. Exclusion criteria were patients with coronary heart disease (angina, myocardial infarction), stroke, diabetes, or acute cardiac, hepatic or renal dysfunction (contraindications for chemotherapy or CEUS), macrocalcific carotid plaque (to avoid attenuation artifacts).

Finally 102 patients with incidental soft carotid plaques (68 males and 34 females, 62.3 ± 12.4 years) were enrolled and randomly divided into two treatment groups: (1) NP (vinorelbine + cisplatin) treatment group (51 cases, 62.1 ± 9.7 years); (2) ENP (Endostar + vinorelbine + cisplatin) treatment group (51 cases, 62.7 ± 14.1 years). In addition, thirty-four subjects (26 males and 8 females, 63.4 ± 10.6 years) in whom soft carotid plaques were detected on carotid ultrasound screening who were not scheduled for chemotherapy (no NSCLC) were enrolled as controls (Figure 1).

### Treatment

The NP group was treated with vinorelbine (Hubei HONCH Pharmaceutical Co., Ltd. China) 25 mg/m^2^ in 40 ml of normal saline by intravenous bolus injection on days 1 and 8 and cisplatin (ShanDong JiuLong Pharmacy Company, China) 30 mg/m^2^ by intravenous infusion on days 1 and 3. This regimen was repeated every 21 days. The ENP group received the same vinorelbine and cisplatin chemotherapy regimen as the NP group but with the addition of daily Endostar (Shandong Harbinger Magenta, Tianjin Pharmaceutical Co., Ltd. P.R. China) treatment at a dose of 7.5 mg/m^2^ by intravenous infusion from day 1 to 14 every 3 weeks. All patients were given routine antiemetic and diuretic treatments during chemotherapy.

### Standard and contrast-enhanced carotid ultrasound imaging

Standard and contrast-enhanced carotid ultrasound imaging was performed with an Acuson Sequoia 512 ultrasound unit using an 8–14 MHz 15L8w linear array probe. The carotid plaque CEUS examinations were performed using a standard imaging protocol at recruitment (baseline) and at 1 month after one cycle treatment for the NSCLC patients by a trained vascular radiologist (YZ with 11 years experience in CEUS). Longitudinal and transverse scanning was performed to examine the carotid arteries with the patient in a supine position to expose the neck. The thickest plaque on the carotid wall with transverse cross section was selected and measured for thickness. The subjects was then scanned with CEUS imaging by switching to contrast pulse sequence (CPS) imaging mode with a low mechanical index of 0.32. The dynamic range was set at 75 dB. The ultrasound probe was held steady and remained at the same position for the selected plaque throughout the examination. The contrast agent SonoVue® (Bracco SpA, Milan, Italy) used in this study was supplied as a lyophilized powder, which was reconstituted by adding 5 ml of 0.9% saline and gently shaking the vial by hand to form a homogeneous microbubble suspension. A 19-gauge cannula was inserted into an antecubital fossa vein and 2.4 mL of SonoVue was injected as a bolus followed by 10 mL of saline flush for each contrast study. A three-way tap was used so that the saline flush could be given immediately after the contrast agent injection. A timer on the sonographic unit was activated at the beginning of the injection. A real time contrast-enhanced cine-loop (at least 3 minutes long) of the carotid plaque was acquired following the injection and stored digitally on magnetic optical disks for later offline analysis. One month later (after first treatment for the patients) all subjects received carotid plaque CEUS again, and all the above scanner settings were kept constant.

### Image analysis

One board-certified vascular radiologist (CZ with 11 years experience) reviewed the cine loops of all the plaques off-line using auto-tracking contrast quantification (ACQ) analysis software (Axius, Siemens Medical Solution, Inc., USA). He was blinded to the clinical and imaging information (such as the timing of the examinations and the groups, whether patients or controls) at the time of the analyses.

**Figure 4:**
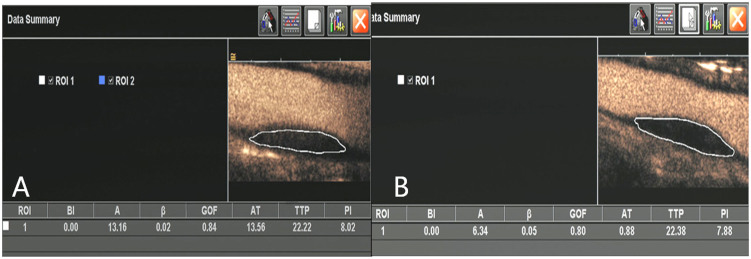
A freehand region of interest (ROI) was drawn around the margin of the plaque using an electronic-cursor, avoiding the lumen and surrounding tissue. Perfusion indices such as peak intensity (PI) and baseline intensity (BI) were calculated by the ACQ software. Enhanced intensity (EI, EI = PI − BI) was reduced from 8.02 dB at baseline (A) to 7.88 dB after treatment (B).

A freehand region of interest (ROI) was drawn around the margin of the plaque using an electronic-cursor, avoiding the lumen and surrounding tissue. The ROI was adjusted manually frame-by-frame as necessary. A time-intensity curve (TIC) for the selected ROI was derived automatically by the scanner software and perfusion indices such as peak intensity (PI) and baseline intensity (BI) were calculated by the ACQ software, and a goodness-of-fit above 0.75 was considered acceptable (this described the discrepancy between the observed and expected values under the logarithmic compression model used by the ACQ software)^31^ (Figure 4). All measurements were performed three times, and the means of these three measurements were calculated and compared. Two of the perfusion indices, PI and BI were used to calculate the enhancement intensity (EI) in the plaque using the formula EI = PI − BI.

### Statistical analysis

Statistical analysis was performed using SPSS software version 13.0 (IBM, USA). Data are expressed as means ± SD. The Shapiro-Wilk test was applied to test for normality. The data among the three groups was compared by one-way analysis of variance (ANOVA) when the data showed a normal distribution. Data between each group at baseline and at 1 month after treatment was compared using the paired Student's t-test. The Mann-Whitney U-nonparametric test was used when the data had a non-normal distribution. The chi-square analysis was used for gender percentage comparison. A *P* < 0.05 was considered to be statistically significant.

## Author Contributions

P.H. designed this study. Z.P., Y.W., Y.Z. and H.H. acquired the data. Y.H., C.L., S.C., P.G. and J.H. interpreted the data. P.H. wrote the main manuscript text. All authors reviewed the manuscript.

## Figures and Tables

**Table 1:** General characteristic of patients

		Before treatment	After treatment	N	T value	P value
**EF (%)**	Control	63.5 ± 5.8	64.1 ± 7.8	34	0.360	0.720
	NP	63.7 ± 7.2	65.3 ± 5.2	48	2.028	0.445
	ENP	64.3 ± 8.6	63.4 ± 6.8	43	0.538	0.592
**SBP (mmHg)**	Control	120.4 ± 13.3	122.1 ± 11.7	34	0.889	0.377
	NP	117.6 ± 11.5	119.4 ± 14.4	48	1.053	0.295
	ENP	118.9 ± 9.1	121.8 ± 16.2	43	1.023	0.309
**DBP (mmHg)**	Control	76.7 ± 8.6	73.5 ± 6.4	34	1.741	0.086
	NP	74.9 ± 6.3	76.3 ± 7.1	48	1.022	0.310
	ENP	75.2 ± 7.8	77.1 ± 5.2	43	1.329	0.187
**TG (mmol/L)**	Control	1.39 ± 0.44	1.38 ± 0.62	34	0.077	0.939
	NP	1.32 ± 0.53	1.35 ± 0.47	48	0.293	0.770
	ENP	1.41 ± 0.51	1.39 ± 0.43	43	0.197	0.845
**HDL (mmol/L)**	Control	2.52 ± 0.48	2.54 ± 0.62	34	0.149	0.882
	NP	2.43 ± 0.42	2.47 ± 0.65	48	0.358	0.721
	ENP	2.47 ± 0.51	2.39 ± 0.44	43	0.779	0.438
**LDL (mmol/L)**	Control	1.24 ± 0.51	1.28 ± 0.48	34	0.3330	0.740
	NP	1.19 ± 0.63	1.23 ± 0.52	48	0.339	0.735
	ENP	1.21 ± 0.57	1.23 ± 0.43	43	0.184	0.855

Note: Data are the mean ± standard deviation. NP = Vinorelbine + Cisplatin; ENP = Endostar + Vinorelbine + Cisplatin; EF = Ejection fraction; SBP = Systolic blood pressure; DBP = Diastolic blood pressure; TC = Total cholesterol; TG = Total triglyceride; LDL = Low-density lipoprotein; HDL = High-density lipoprotein.

There were no significant differences in EF, blood pressure, serum lipids among the three groups at recruitment (*P* > 0.05 for all). There were no significant changes in any group in, EF, blood pressure and blood lipid levels at recruitment and 1 month after treatment (*P* > 0.05 for all).

**Table 2:** Effect of Endostar on plaque thickness and neovascularization

		Before treatment	After treatment	N	T value	P value
**Plaque thickness (mm)**	Control	2.38 ± 0.49	2.41 ± 0.72	34	0.201	0.841
	NP	2.42 ± 0.63	2.39 ± 0.71	48	0.219	0.827
	ENP	2.41 ± 0.57	2.39 ± 0.62	43	0.156	0.877
**EI (dB)**	Control	7.67 ± 1.44	7.69 ± 1.33	34	0.060	0.953
	NP	7.65 ± 1.25	7.59 ± 1.76	48	0.193	0.848
	ENP	7.78 ± 1.56	6.23 ± 1.36	43	4.91	<0.01

Note: Data are the mean ± standard deviation. NP = vinorelbine + cisplatin; ENP = Endostar + vinorelbine + cisplatin; EI = Enhanced intensity.

There were no significant differences in plaque thickness and EI among the three groups at recruitment (*P* > 0.05 for all). There was no significant change in the EI of plaque in the controls or NP groups at 1 month after treatment (*P* > 0.05). While EI in the ENP group was significantly reduced at 1 month after treatment (*P* < 0.01) and significantly lower than that in the controls or NP groups at 1 month after treatment (*P* < 0.001 for both).
